# Discovery and mapping of a new gene in wheat for race-specific adult plant resistance to *Puccinia striiformis* f. sp. *tritici*

**DOI:** 10.1007/s00122-026-05311-9

**Published:** 2026-08-05

**Authors:** R. F. Park, M. Chhetri, K. S. Sandhu, L. Ziems, D. Singh

**Affiliations:** Sydney Institute of Agriculture, Plant Breeding Institute Cobbitty, University of Sydney, PMB 4011, Sydney, NSW 2567 Australia

## Abstract

**Abstract:**

Key message

A non-durable adult plant stripe rust resistance gene in wheat was mapped to the distal region of chromosome 2DS and designated *Yr88*.

**Abstract:**

Wheat stripe (yellow) rust, caused by *Puccinia striiformis* f. sp. *tritici* (*Pst*), is one of the most damaging fungal pathogens of wheat globally. Efforts to breed improved wheat cultivars have placed high priority on incorporating resistance to *Pst*. The resistances used are expressed at either all growth stages (all stage resistance; ASR) or at post-seedling growth stages (aka adult plant resistance; APR). Although APR has a reputation for being more durable than ASR examples have been documented of virulence for this type of resistance in the UK, Europe and in North America. Incursions of two genetically and pathogenically distinct pathotypes of *Pst* into Australia in 2017 and 2018 changed adult plant field stripe rust responses of many wheat cultivars grown in eastern Australia at that time. In some cases, changes in response could not be accounted for based on virulence on ASR genes, suggesting that the protective value of APR may have changed. This study reports the discovery of a new gene conferring race-specific APR to *Pst* pathotype 198 E16 A- J+ T+ 17+, a member of the internationally accepted multi-locus genotype (MLG) PstS13. The gene was mapped to the distal region of chromosome 2DS and designated *Yr88*. Evidence is provided for the presence of *Yr88* in near isogenic genes carrying selected ASR and APR *Yr* genes, four Australian wheat cultivars (LRPB Hellfire, Sherriff CL Plus, Vixen, Wyalkatchem) plus the Mexican green revolution wheat cultivar Lerma Rojo 64 and the Pakistani wheat cultivar Moomal 2000.

**Supplementary Information:**

The online version contains supplementary material available at 10.1007/s00122-026-05311-9.

## Introduction

A survey conducted in 2016/2017 estimated that global production of the five most important crops (wheat, rice, maize, potato, soybean) was reduced by 21.5% due to pests and pathogens (Savary et al. 2019). This study ranked wheat stripe (yellow) rust, caused by *Puccinia striiformis* Westend f. sp. *tritici* Erikss. (*Pst*), among the top eight most damaging biotic stress agents globally. Beddow et al. (2015) estimated that 88% of the world’s wheat crops were vulnerable to *Pst* infection, with annual loss estimates of 5.47 million tons of wheat worth US$979 million. Individual crop losses due to stripe rust have been documented as high as 100% (Wellings 2011; Chen 2020).

The impact of diseases such as stripe rust in many of the world’s wheat growing regions has been reduced significantly by the use of genetic resistance. In 2009, resistance to stripe rust in wheat was estimated to save $431 million per year in Australia (Murray and Brennan 2009). Rust resistance in cereals is often defined based on growth stage at which it is expressed: All stage resistance (ASR; aka seedling resistance) is expressed at all growth stages from primary leaf emergence onwards; adult plant resistance (APR) is expressed at post-seedling growth stages only. ASR is also known as major gene resistance, and APR often as minor gene resistance, due to differences in the phenotypic effect of each. Different types of APR have been described based on the time at which resistance is first detected (e.g., Park and Rees 1989; Cromey 1992; Ma and Singh 1996; Norman et al. 2023). To date, 87 loci governing resistance to *Pst* have been catalogued in wheat (Sharma et al. 2024), of which 61 are regarded as conferring ASR and 25 as conferring APR. Most of the catalogued ASR genes conferring resistance to *Pst* that have been used in agriculture have proven to be race-specific, with pathotypes carrying matching virulence being detected soon after deployment.

Although some APR genes have shown a greater tendency to be more durable, they can be less effective at protecting against yield loss under high disease pressure (Bariana and McIntosh 1995; McIntosh et al. 1995; Ma and Singh 1996). Virulence has been documented for some sources of APR to stripe rust in wheat. Stubbs (1985) reported that uncharacterized APR to stripe rust in the cultivars Dippes Triumph and Alba were overcome 3 years and 7 years after release, respectively. Johnson (1992) summarized research undertaken in the UK that characterized race-specific APR to *Pst* in European wheats that was overcome in the 1970 s and resulted in extensive stripe rust epidemics. Based on isolate specificity, four APRs were identified as being race specific, and although none were mapped to chromosomes, they were given the locus designations *Yr11* through *Yr14* (McIntosh et al. 1995). Studies that investigated interactions between 12 wheat cultivars grown in eastern USA and isolates of *Pst* collected between 1990 and 2013 by Milus et al. (2015) similarly found race-specific APR, with six major virulence patterns being identified among the isolates tested. The present study reports the discovery of a gene conferring race-specific APR to *Pst* pathotype 198 E16 A- J + T + 17 +, a member of the internationally accepted multi-locus genotype (MLG) PstS13. The gene was mapped to the distal region of chromosome 2DS and designated *Yr88*. Evidence is provided for its presence in several Australian and international wheat cultivars.

## Materials and methods

### Wheat germplasm

All genotypes investigated were spring wheats. Plants were grown in 9-cm-diameter plastic pots with either four (seedling) or two (all other growth stages) lines/pot. A potting mix comprising composted pine bark and coarse sand in a ratio of 4:1 was used and pots were fertilized with a soluble nitrogenous fertilizer Aquasol® (Hortico Pty Ltd, Australia) at the rate of 30 g in 10 L of water for 200 pots prior to sowing and with a second application at 3 weeks for older plants. Plants were maintained both before and after inoculation in a temperature-controlled greenhouse microclimate room (17–18 ± 1.5 °C). All experiments were conducted under natural lighting during September/October 2023 at the Plant Breeding Institute Cobbitty, when daylength varied from 11.5 to 13.5 h. Differential sets comprising 32 wheat stripe rust differential genotypes (Online Resource Table S1) inoculated 8–9 days after sowing were included in all seedling and adult plant growth stage tests to check the purity and identity of the pathotypes used in each.

### Pathogen isolates, inoculation and phenotypic assessments

Reference isolates of 14 Australian pathotypes (pts) of *Pst* were used (Table 1), and all were first identified in routine national race surveys conducted by staff at the University of Sydney Plant Breeding Institute and are maintained in liquid nitrogen. Each reference isolate is assigned a unique culture number when accessioned in the University of Sydney cereal rust collection, and this culture number is used here to refer to each of the pathotypes for simplicity. Four of the currently accepted 19 MLGs (Hovmøller et al. 2025) are represented by the isolates, namely PstS0, PstS1, PstS10 and PstS13.

**Table 1:** Pathotypes of *Puccinia striiformis* f. sp. *tritici* used in all stage resistance gene postulation and/or adult plant response tests

Culture number	Pathotype/race	Year	Virulence phenotype*	Genetic group
Pst415	104 E137 A-	1982	-,2,3,4,-,-,-,-,-,-,-,-,-,25,-,-,-,S92/O,-,AvR,AvS,-,-	Not tested
Pst419	108 E141 A-	1983	-,2,3,4,-,6,-,-,-,-,-,-,-,25,-,-,-,S92/O,-,-,AvS,-,-	PstS0
Pst420	108 E141 A +	1983	-,2,3,4,-,6,-,-,-,-,-,-,-,25,-,-,-,S92/O,-,-,AvR,AvS,-,-	PstS0
Pst444	110 E143 A +	1986	-,2,3,4,-,6,7,-,-,-,-,-,-,25,-,-,-,S92/O,-,-,AvR,AvS,-,-	PstS0
Pst544	104 E137 A- 17 +	1999	-,2,3,4,-,-,-,-,-,-,-,17,-,25,-,-,-,S92/O,-,-,AvS,-,-	PstS0
Pst620	64 E0 A-	2009	-,-,-,4,-,-,-,-,-,-,-,-,-,-,-,-,-,S92/O,-,-,AvS,-,-	PstS0
Pst572	134 E16 A +	2002	-,2,-,-,-,6,7,8,9,-,-,-,-,25,-,-,-,-,-,AvR,AvS,-,-	PstS1
Pst598	150 E16 A +	2005	-,2,-,-,-,6,7,8,9,10,-,-,24,25,-,-,-,-,-,AvR,AvS,-,-	PstS1
Pst599	134 E16 A + 17 +	2006	-,2,-,-,-,6,7,8,9,-,-,17,-,25,-,-,-,-,-,AvR,AvS,-,-	PstS1
Pst617	134 E16 A + 17 + 27 +	2010	-,2,-,-,-,6,7,8,9,-,-,17,-,25,27,-,-,-,-,AvR,AvS,-,-	PstS1
Pst674	239 E237 A- 17 + 33 +	2017	1,2,3,4,-,6,7,-,9,-,-,17,-,25,-,32,33,S92/O,SP,-,AvS,-,-	PstS10
Pst687	198 E16 A- J + T + 17 +	2018	-,2,-,4,-,6,7,8,9,-,-,17,-,-,-,32,-,-,-,AvR,AvS,J,T	PstS13
Pst698	238 E191 A- J + T + 17 +	2021	-,2,-,4,-,6,7,8,9,-,-,17,-,25,-,32,-,-,-,AvR,AvS,J,T	Not tested
Pst699	238 E191 A + 17 + 33 +	2021	-,2,3,4,-,6,7,8,9,-,-,17,-,25,-,32,33,S92/O,-,AvR,AvS,-,-	Not tested

^*^Numbers and symbols indicate virulence and avirulence (-) corresponding to stripe rust resistance genes: *Yr1*, *Yr2*, *Yr3*, *Yr4*, *Yr5*, *Yr6*, *Yr7*, *Yr8*, *Yr9*, *Yr10*, *Yr15*, *Yr17*, *Yr24*, *Yr25*, *Yr27*, *Yr32*, *Yr33*, Suwon 92/Omar (S92/O), Spaldings Prolific (SP), Avocet R (AvR), Avocet S (AvS), *YrJackie* (J), *YrTobruk* (T)

Inoculations were performed using urediniospores suspended in light mineral oil (Isopar L®) at the rate of about 1 mg/1 ml and dispersed by misting with an airbrush above plants to allow most oil to evaporate and urediniospores to settle on plants. To prevent contamination, all equipment was washed in 70% ethanol and then running tap water between inoculations. Inoculated seedlings were exposed to artificial dew generated by a humidifier (Contronics HU-25 Ultrasonic Humidifier) and reverse osmosis water supply system (Condair RO-HM) for 15 h of misting in darkness at 10–12 °C.

Disease response was assessed once the susceptible control genotype Morocco had reached full susceptibility, using a modified "0"–"4" infection type (IT) scale as described by McIntosh et al. (1995). Variations in IT were indicated by the use of "-" (less than average for the class), "+" (more than average for the class), "C" (chlorosis) and "N" (necrosis).

### Effect of growth stage on resistance to *Pst* in Morocco, Avocet S and two Avocet S near isogenic Lines (NILs) with catalogued APR genes

The first experiment assessed the response of four genotypes, Morocco, a selection of the Australian wheat cultivar Avocet known as Avocet S and two lines near isogenic to Avocet S carrying the catalogued APR genes *Yr18* and *Yr29* (viz. Avocet S + Yr18, and Avocet S + Yr29), inoculated at four growth stages. Seed of each line and the susceptible control Morocco was sown on four dates to enable a single inoculation of plants that were 8 days (Zadoks Growth Stage (ZGS) 11; Zadoks et al. 1974), 14 days (ZGS 12), 22 days (ZGS 13) or 29 days (concurrent ZGS 14, 20) old at the time of the inoculation. Seven pathotypes of *Pst* were used.

### Effect of growth stage on resistance to *Pst* in Morocco, Avocet R, Avocet S, and seven Avocet S NIL stocks

Multi-pathotype stripe rust assays were conducted on Morocco, Avocet R, Avocet S, and seven NIL stocks in the Avocet S background. Five of the NILs carried single ASR genes (*Yr6*, *Yr7*, *Yr8*, *Yr9* and *Yr17*) and two carried single APR genes (*Yr18* and *Yr29*). The lines were sown on three dates to allow a single inoculation of plants that were 8 days (ZGS 11), 21 days (ZGS 13) or 28 days old (concurrent ZGS 14, 20) at the time of inoculation. Five pathotypes of *Pst* were used.

### Genetic analysis of the adult plant response of Avocet R to *Pst*

A doubled haploid (DH) population based on the cross Teal (AGG12330)/Avocet R plus the parents were assessed at post-seedling growth stages for response to two pathotypes of *Pst*, Pst419 (pt. 108 E141 A-) and Pst687 (pt. 198 E16 A- J + T + 17 +). A total of 77 lines were used, all of which were included in the 92 lines from the same population used in a previous study to map the *Yr73* + *Yr74* complementary resistance in Avocet R (Dracatos et al. 2015). The plants were inoculated 28 days (concurrent ZGS 14, 20) after sowing.

### Genetic analysis of APR to *Pst* in Avocet S and marker trait mapping of APR

A recombinant inbred population (F_7:8_) of 95 progeny was developed from the cross of Kazouria Taliani by Avocet S. The population and parents were assessed for response to Pst687 at post-seedling growth stages, being inoculated at 28 days (concurrent ZGS 14, 20) after sowing. Phenotypic assessments were performed independently by two assessors. A line that comprised individuals with two different phenotypes was classified as segregating and the number of individuals within each phenotypic class were recorded, for example: 12 Plants (P) “;N”, 2P “3+ ”. Fourteen such segregating lines were recovered and excluded from the analysis.

Genomic DNA was extracted from single plants of each recombinant inbred line (RIL) and the parents. Extractions were made from young leaf tissue using a modified CTAB method (Bansal et al. 2014) and quantified before genotyping. A targeted genotype by sequencing (tGBS) assay was used to genotype each line. This system is based on an Illumina 40 K XT-chip designed using exon-based SNPs from a 90 K array (www.latrobe.edu.au/agribio). Samples were processed by AgriBio using a custom bioinformatics pipeline that processes sample reads from the tGBS assay to generate genotype calls for polymorphic loci.

For QTL mapping in the biparental population, ITs were converted to binary scores of 1 for lines that were susceptible (IT “3+”) and 0 for lines that were resistant (IT < "3+”). The mapping was performed in R using the "statgenMPP" package, which applies an identity-by-descent (IBD)-based mixed model framework (Li et al. 2022). Genotypes were derived from raw tGBS genotype calls and following filtering and formatting for statgenMPP, 1,959 tGBS markers were used for QTL mapping. IBD probabilities were calculated at 5 Mbp intervals across the physical map of the IWGSC v1.0. Genome wide scans were conducted using a multi-round procedure in which the most significant locus was added as a cofactor in the successive model until no additional loci exceeded the significance threshold. A 10 Mbp window was applied to prevent the detection of closely linked cofactors. Loci with a -log10p ≥ 4 were declared QTL. For each significant region, the proportion of phenotypic variance explained and parental allele effects were estimated, and the parent contributing the favorable allele was identified. The QTL mapping and marker frequency analyses were based on the same genotypic dataset but used different processed marker outputs.

For bulked-segregant marker frequency analysis, 5,125 genotype calls recoded according to parental origin were provided by AgriBio, with Avocet S alleles coded as 0 and Kazouria Taliani alleles coded as 1. Lines were grouped into resistant and susceptible bulks based on binary IT scores. For each marker, the mean allele score of the resistant group and the mean allele score of the susceptible group were calculated, and discriminant value was computed as (μ_susceptible_−μ_resistant_)^2^ (Wenzl et al. 2006, 2007). This quantitative measure represents the level of allelic discrimination between the two classes with larger values indicating stronger segregation and a maximum possible value of 1.0 denoting complete (100%) separation of parental alleles. Markers were ranked by this value to identify loci with the greatest allele frequency difference between bulks. Only these perfectly segregating markers are reported here, as they represent the strongest possible marker–trait associations.

Marker Xgwm102, reported as a flanking marker of an APR locus on chromosome 2DS putatively corresponding to *Yr16* (Agenbag et al. 2012) was projected on to IWGSC v1.0 reference to provide positional comparison with the chromosome 2D region identified in this study. To anchor the SSR marker Xgwm102, the GrainGenes database (Yao et al. 2022) was queried to obtain WMS102 probe report, which contains the published primer sequences (Röder et al. 1998). Each primer was aligned using BLASTn (v2.12.0 +) via the WheatOmics 1.0 interface (Ma et al. 2021), restricting the search to chromosome 2D and using default nucleotide BLAST parameters. Perfect matches were accepted to define the marker position. No probe, primer or sequence information could be identified for the second reported flanking marker wPt-664520. Because the published *Yr16* markers are linked rather than gene specific and were not present in the Kazouria Taliani/Avocet S genotypic data, haplotype comparison across the reported *Yr16* interval was not possible.

QTL regions identified through Kazouria Taliani/Avocet S biparental mapping and marker frequency analysis as well as projected *Yr16* associated marker Xgwm102 were visualized on the IWGSC v1.0 reference genome with Software MapChart 2.32 (Voorrips 2002).

### Seedling and adult plant stripe rust responses of selected wheat cultivars

A set of 82 Australian wheat cultivars and 10 additional wheat genotypes (Online Resource Table S2) were tested for stripe rust response at seedling growth stages and at post-seedling growth stages. Seedling tests were conducted by inoculating plants 9 days (ZGS 11) after sowing. Twelve pathotypes of *Pst* were used, which allowed assessments of ASR and in many cases postulations of ASR genes present in each cultivar. Five of these pathotypes were then used to assess post-seedling response of the same set of wheats. In these tests, plants were inoculated 26 days after sowing (concurrent ZGS 14, 20). All lines were also genotyped with markers linked to one of three genes conferring APR to *Pst*: marker csLV34 (Lagudah et al. 2006), linked to *Yr18*/*Lr34*/*Sr57*; marker csLV46G22 (Ren et al. 2017) linked to *Yr29*/*Lr46*/*Sr58*; marker TM4 linked to *Yr46*/*Lr67*/*Sr55* (Moore et al. 2015).

## Results

### Effect of growth stage on resistance to *Pst* in Morocco, Avocet S and two Avocet S NILs with catalogued APR genes

The genotypes Morocco, Avocet S, Avocet S + Yr18 and Avocet S + Yr29 were inoculated with seven pathotypes of *Pst* 8 days, 14 days, 22 days and 29 days after sowing. Morocco was susceptible to all pathotypes at all growth stages (Table 2). Avocet S and the two Avocet S Nil stocks were fully susceptible to each of the seven pathotypes when inoculated 8 days after sowing, indicating no effective ASR (Table 2), and all were susceptible to pathotypes Pst674 and Pst699 at all four growth stages, indicating no effective APR (Table 2). Plants of the three Avocet S genotypes inoculated 14 days after sowing were susceptible to the remaining five pathotypes, although some resistance was apparent in those challenged with pathotypes Pst687 and Pst698. Low to high levels of resistance were recorded in NILs carrying *Yr18* and *Yr29* that were inoculated with pathotypes Pst620, Pst444 or Pst572, 22 days and 29 days after sowing. Uniformly high levels of resistance were recorded for all three genotypes inoculated 22 days and 29 days after sowing with pathotypes Pst687 and Pst698. These results demonstrated clearly that Avocet S and the two lines near isogenic to it carry uncharacterized APR that is effective only to pathotypes Pst687 and Pst698, which became effective between 15 and 22 days after sowing. The resistance detected was given the temporary designation *YrAvSAPR*.

**Table 2:** Infection type response of Morocco, Avocet S and two lines near isogenic to Avocet S carrying adult plant stripe rust resistance genes *Yr18* or *Yr29* to seven Australian pathotypes of *Puccinia striiformis* f. sp. *tritici* at four growth stages

		Pathotype [culture number]						
	Age at	64 E0 A-	110E143 A +	134 E16 A +	238 E191 A- J + T + 17 +	198 E16 A- J + T + 17 +	239 E237 A- 17 + 33 +	238 E191 A + 17 + 33 +
Genotype	inoculation	[Pst620]	[Pst444]	[Pst572]	[Pst698]	[Pst687]	[Pst674]	[Pst699]
Morocco	8 days	3+	3+	3+	3+	3+	3+	3+
	14 days	3+	3+	3+	3+	3+	3+	3+
	22 days	3+	3+	3+	3+	3+	3+	3+
	29 days	3+	3+	3+	3+	3+	3+	3+
Avocet S	8 days	3+	3+	3+	3+	3+	3+	3+
	14 days	3+	3+	3+	3CN	33+CN	3+	3+
	22 days	33+CN	3+	33+C	0;-	0;-	3+	3+
	29 days	3C	3C	33+	0;-	0;-	3+	3+
Avocet S + Yr18	8 days	3+	3+	3+	3+	3+	3+	3+
	14 days	3+	3+	3+	3C	2+C	3+	3+
	22 days	3C	3+	3+	0;=	0;=	3+	3+
	29 days	3C	3+	2++3C	0;=	0;-	3+	3+
Avocet S + Yr29	8 days	3+	3+	3+	3+	3+	3+	3+
	14 days	3+C	3+	3CN	;12+CN	;12+CN	3+	3+
	22 days	3C	3+	2+C	0	0	3+	3+
	29 days	3C	33+	;+C	0;-	0;-	3+	3+

### Effect of growth stage on resistance to *Pst* in Morocco, Avocet R and seven Avocet S NIL stocks

The potential presence of *YrAvSAPR* was assessed in Avocet R and seven lines near isogenic to Avocet S carrying single catalogued ASR (Avocet S + Yr6, Avocet S + Yr7, Avocet S + Yr8, Avocet S + Yr9, Avocet S + Yr17) or APR (Avocet S + Yr18, Avocet S + Yr29) genes. These lines plus the controls Morocco and Avocet S were inoculated with five pathotypes of *Pst* (Pst444, Pst598, Pst674, Pst687, Pst699) at 8 days, 21 days, or 28 days after sowing.

**Fig. 1:**
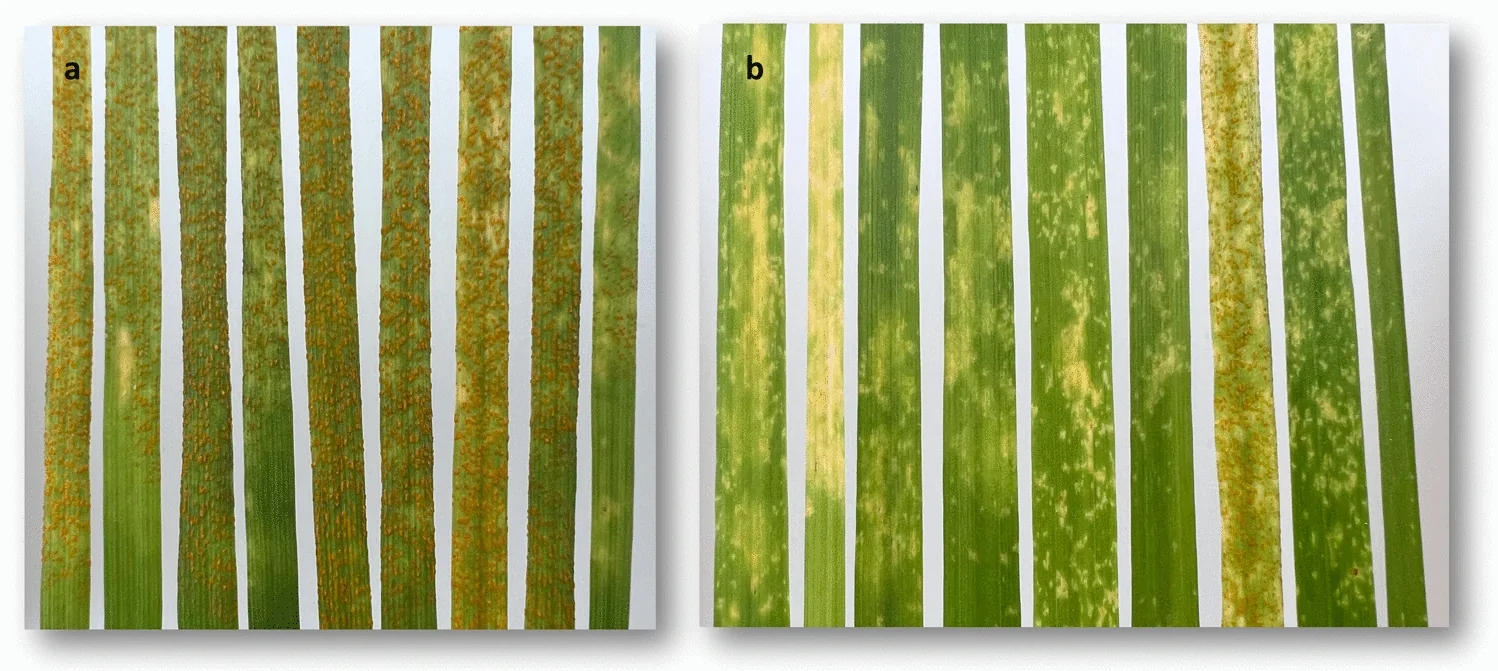
Leaves of (L to R) Avocet S, Avocet R, Avocet S + Yr18, Avocet S + Yr29, Avocet S + Yr6, Avocet S + Yr7, Avocet S + Yr8, Avocet S + Yr9, Avocet S + Yr17 inoculated with *Puccinia striiformis* f. sp. *tritici* pathotype 198 E16 A- J + T + 17 + (Pst687): **a** 8 days after sowing (seedling leaves), **b** 28 days after sowing (fourth leaves)

The control genotype Morocco was susceptible to all five pathotypes at all three growth stages. In tests with Pst687, Avocet S and the five Avocet S ASR NIL lines were susceptible when inoculated 8 days after sowing (Fig. 1a), and all except Avocet S + Yr8 displayed high levels of resistance to Pst687 when inoculated 28 days after sowing (Fig. 1b). These results showed that *YrAvSAPR* was present in Avocet S and all Avocet S NILs except that carrying *Yr8*.

All interactions between the remaining four pathotypes with Avocet S and the Avocet S NILs carrying ASR genes were compatible except where a pathotype was avirulent for the ASR gene present (viz. Pst444, *Yr8*, *Yr9* and *Yr17*; Pst598, *Yr8* (partial) and *Yr17*; Pst674, *Yr8*; Pst699, *Yr8*) (Table 3). Interactions between these four pathotypes and the Avocet S NIL stocks carrying the APR genes *Yr18* or *Yr29* were compatible with the exception of Avocet S + Yr29, resistance in which was apparent on plants inoculated with Pst598 28 days after sowing (Table 3).

**Table 3:** Infection type response of Morocco, Avocet S, Avocet R, and seven lines near isogenic to Avocet S carrying stripe rust resistance genes to five Australian pathotypes of *Puccinia striiformis* f. sp. *tritici* at three growth stages

		Pathotype [culture number]				
	Age at inoculation	110 E143 A +	150 E16 A +	239 E238 A- 17 + 33 +	198 E16 A- 17 + J + T +	238 E191 A + 17 + 33 +
Genotype	inoculation	[Pst444]	[Pst598]	[Pst674]	[Pst687]	[Pst699]
Morocco	8 days	3+	3+	3+	3+	3+
	21 days	3+	3+	3+	3+	3+
	28 days	3+	3+	3+	3+	3+
Avocet R	8 days	3+	3+	12C	3+	3+
	21 days	3+	3+	;1++CN	3CN	3+
	28 days	3+	33+	;++CN	;2=C	3+
Avocet S	8 days	3+	3+	3+	3+	3+
	21 days	3+	3+	3+	3C	3+
	28 days	3+	3+	3+	;1C	3+
Avocet S + Yr6	8 days	3+	3+	3+	3+	3+
	21 days	3+	3+	3+	12C	3+
	28 days	3+	3+	3+	;-	3+
Avocet S + Yr7	8 days	3+	3+	3+	3 +	3+
	21 days	3+	3+	3+	12C	3+
	28 days	3+	3+	3+	;-	3+
Avocet S + Yr8	8 days	;	3CN	0;	3+	12CN
	21 days	;	;+N	;	3CN	12CN
	28 days	0;	;1++CN	0;-	3CN	;++N
Avocet S + Yr9	8 days	;	3+	3+	3+	3+
	21 days	;	3+	3+	12C	3+
	28 days	0;	3+	3+	;	3+
Avocet S + Yr17	8 days	;1+N	3CN	3+	12CN	3+
	21 days	;N	12N	3+	;++C	3+
	28 days	0;	;CN	3CN	0;-	3+N
Avocet S + Yr18	8 days	3+	3+	3+	3+	3+
	21 days	3+	3+	3+	3CN	3+
	28 days	3+	3+C	3+	;+C	3+
Avocet S + Yr29	8 days	3+	3+	3+	3+CN	3+
	21 days	3+	3C	3+	;1++C	3+
	28 days	3N	;1++C	3+	;C	3+

**Fig. 2:**
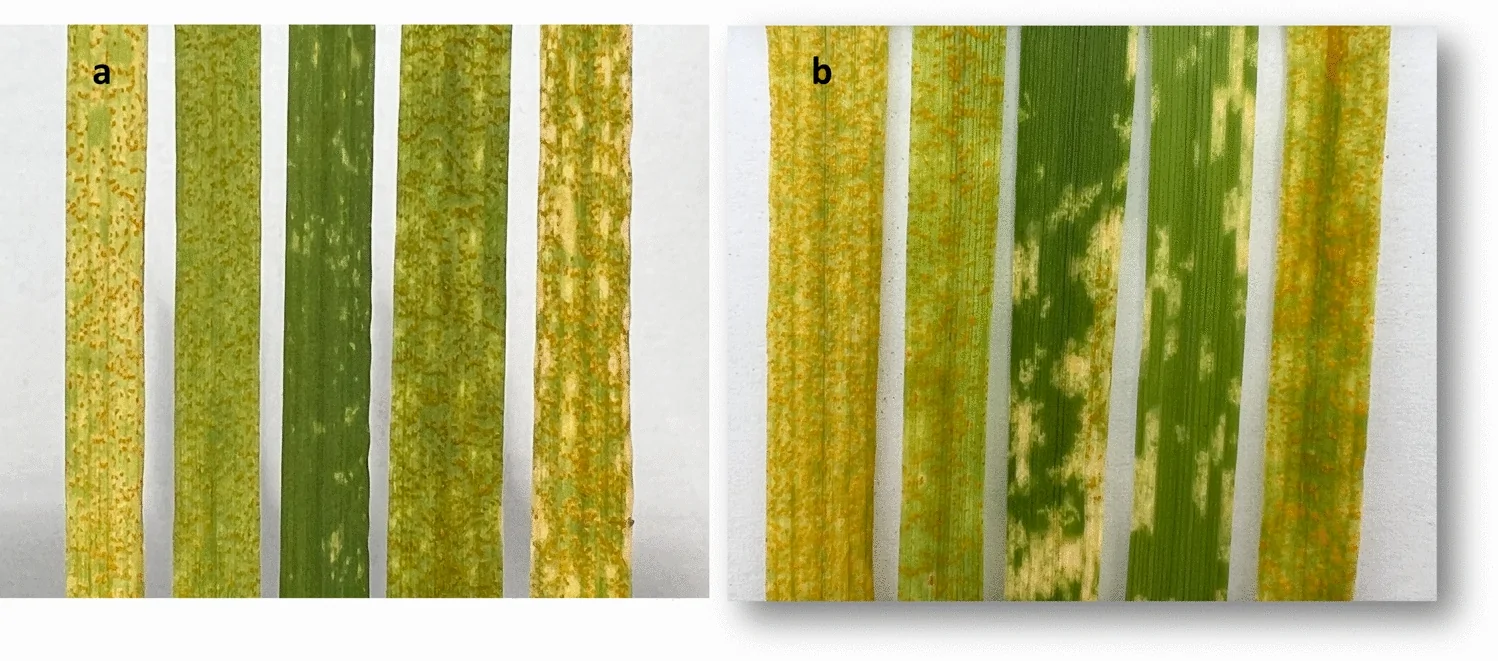
Fourth leaves of **a,** Avocet S and **b**, Avocet R, inoculated with *Puccinia striiformis* f. sp. *tritici* pathotypes (L to R) 110 E143 A + [Pst444], 150 E16 A + [Pst598], 198 E16 A- J + T + 17 + [Pst687], 239 E237 A- 17 + 33 + [Pst674], and 238 E191 A + 17 + 33 + [Pst699] 28 days after sowing

Despite being resistant to Pst687, the response of Avocet R was noticeably less resistant than that of Avocet S and the other Avocet S NIL stocks postulated to carry *YrAvSAPR*, resembling the low infection type seen on this stock when challenged with the *Yr73* + *Yr74*-avirulent pathotype Pst674 (Table 3, Fig. 2a, b), suggesting that this genotype lacked *YrAvSAPR*.

### Genetic analysis of the adult plant response of Avocet R to Pst687

To assess the potential absence of *YrAvSAPR* in Avocet R, a doubled haploid population of 77 lines based on the cross Teal/Avocet R, the two parents, and Avocet S were inoculated with Pst687 and a second pathotype virulent for *YrAvSAPR* but avirulent for the *Yr73* + *Yr74* complementary resistance present in Avocet R (viz. pt. 108 E141 A-, Pst419) 28 days after sowing. One line (Teal/Avocet R 55) was heterogeneous in its response and was therefore excluded (Online Resource Table S3). In tests with pathotype Pst419, Avocet S was susceptible, Avocet R was resistant, Teal was susceptible, and segregation within the progeny was 12 resistant: 64 susceptible (Online Resource Table S3), fitting the segregation model of 1R: 3S expected for two complementary genes (χ^2^ 3.44, *P* > 0.06). In tests with Pst687, Teal was again susceptible and although both Avocet S and Avocet R were resistant, the low infection type response of Avocet R to this pathotype was once again less resistant than that of Avocet S (Online Resource Table S3). Segregation for response to Pst687 among the 77 progeny was exactly the same as that recorded with Pst419 (Online Resource Table S3), indicating that Pst687 is avirulent for the *Yr73* + *Yr74* resistance and that Avocet R lacks the *YrAvSAPR* resistance.

### Genetic analysis of *YrAvSAPR* in Avocet S and marker trait mapping of APR

Previous greenhouse and field studies established that Kazouria Taliani (AGG12911) was seedling susceptible to many Australian pathotypes of *Pst* but carried high levels of APR that were conferred by two APR genes, one of which is likely *Yr46*/*Lr67*/*Sr55* (Kankwatsa et al. 2019). Preliminary tests showed that Kazouria Taliani was susceptible to Pst687 when inoculated 8 days and 28 days after sowing (data not presented). A F_7:8_ RIL population (*n* = 95) derived from the cross Kazouria Taliani/Avocet S plus the two parents were inoculated with Pst687 28 days after sowing. All plants were consistently assigned to the same resistance class by two assessors. Of the 95 RILs, two lines showed very poor germination with weak seedlings and 14 showed a low degree of segregation and were excluded from analyses. Out of the remaining 79 non-segregating RILs, 32 were resistant and 47 susceptible (Online Resource Table S4), fitting the expected segregation of a single gene (1:1, χ^2^ 2.85, *P* > 0.09).

Genotyping was conducted for 72 (30 resistant, 42 susceptible) of the 79 non-segregating lines (Online Resource Table S4). 5,146 polymorphic markers were genotyped in the parents and mapping population, consisting of 1,976 tGBS markers and 3,170 SNP markers. Physical positions on the IWGSC Chinese Spring assembly v1.0 (IWGSC v1.0 2018) were provided for 5,125 markers.

**Fig. 3:**
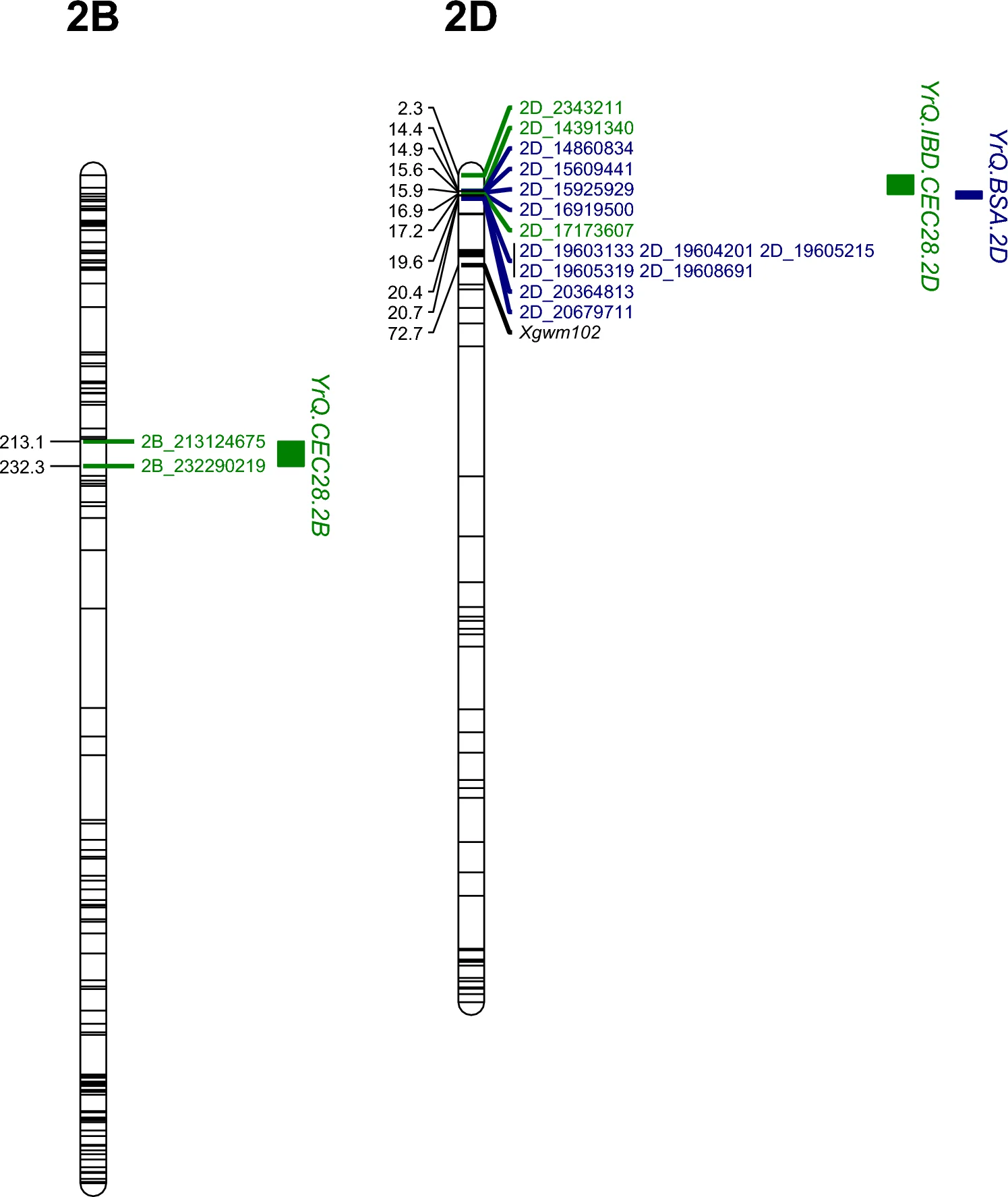
Visualization of IWGSC Chinese Spring assembly v1.0 chromosomes 2B and 2D showing QTL associated with stripe rust response of 95 F_7:8_ RIL progeny from the cross Kazouria Taliani/Avocet S inoculated with *Puccinia striiformis* f. sp. *tritici* pathotype Pst687 (198 E16 A- J + T + 17 +) 28 days after sowing under greenhouse conditions. Green font indicates QTL and associated markers; blue font indicates SNPs that segregated perfectly by bulked-segregant marker frequency analysis; and black font indicates the projected SSR marker Xgwm102 previously reported as flanking *Yr16*

QTL mapping of the stripe rust response of plants inoculated 28 days after sowing detected a major locus on chromosome 2D and a minor locus on 2B (Online Resource Fig. S1, Fig. 3). The 2D QTL was the dominant signal, explaining approximately 86% of the phenotypic variance, with a peak near 14.39 Mbp (− log10P 31.1). The secondary minor QTL on chromosome 2B accounted for about 9% of the variance (peak at 232.29 Mbp, − log10P 4.58), with the resistance allele contributed by Kazouria Taliani (Online Resource Table S5).

**Fig. 4:**
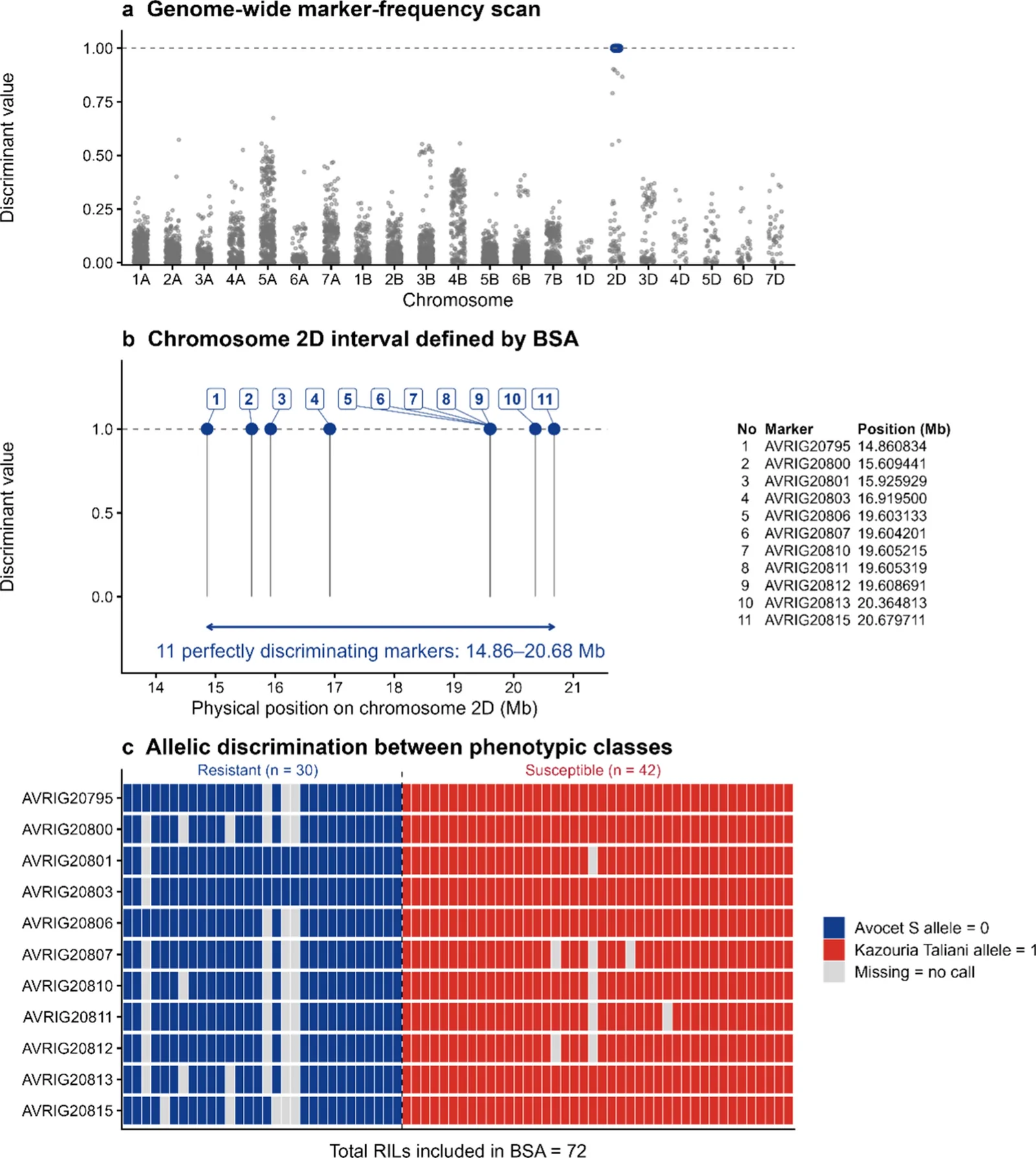
Bulked-segregant marker frequency analysis of the stripe rust response of 72 genotyped F_7:8_ RIL progeny from the cross Kazouria Taliani/Avocet S inoculated with *Puccinia striiformis* f. sp.* tritici* pathotype Pst687 (198 E16 A- J + T + 17 +) 28 days after sowing under greenhouse conditions. **a** Genome wide marker frequency scan showing discriminant values across chromosomes; **b** chromosome 2D region containing 11 markers that perfectly discriminated resistant and susceptible progeny, spanning 14.86–20.68 Mb on the IWGSC Chinese Spring v1.0 reference; **c** genotype pattern of the 11 chromosome 2D markers across resistant and susceptible progeny. Blue indicates the Avocet S allele, red indicates the Kazouria Taliani allele, and gray indicates missing genotype calls

Eleven markers on chromosome 2D showed discriminant value of 1.0 indicating complete (100%) segregation of parental alleles between resistant and susceptible lines. All resistant lines carried the Avocet S allele and all susceptible lines carried the Kazouria Taliani allele. These markers span positions 14.86 Mbp to 20.68 Mbp on the IWGSC v1.0, defining a discrete region of approximately 5.8 Mbp that provides unequivocal discrimination between phenotypic classes (Fig. 4; Online Resource Table S5).

The region on chromosome 2D was confirmed by bulked-segregant marker frequency analysis (Fig. 3, Fig. 4). To compare the chromosome 2D region identified here with the previously reported *Yr16* locus, SSR marker Xgwm102 was anchored to the IWGSC v1.0 reference genome. BLAST alignment of the published primer sequences placed Xgwm102 at ~ 72.66 Mb on chromosome 2D, outside the 14.86–20.68 Mb interval defined by marker frequency analysis in this study (Fig. 3). This positional comparison, together with the distinct infection type and pathotype specificity of the resistance detected here, supports the conclusion that *Yr88* is distinct from *Yr16*.

### Seedling and adult plant stripe rust responses of selected Australian wheat cultivars

A set of 82 Australian wheat cultivars and 10 control genotypes were subjected to seedling tests (inoculated 8 days after sowing) with 11 pathotypes of *Pst* to characterize the ASR present in each and to postulate the ASR gene(s) where possible. The same panel of cultivars was then tested at the post-seedling growth stage (inoculated 26 days after sowing) with five pathotypes. All were marker genotyped for the APR genes *Yr18*/*Lr34*/*Sr57*, *Yr29*/*Lr46*/*Sr58* and *Yr46*/*Lr67*/*Sr55.*

In seedling tests, Avocet R was resistant to the three *Yr73* + *Yr74*-avirlent pathotypes (viz*.* Pst415, Pst620, Pst687) and the remaining nine control genotypes were susceptible to all 12 pathotypes. Nine cultivars were susceptible to all 11 pathotypes, indicating that they lacked ASR genes detectable with the array of cultures used (Online Resource Table S6). The catalogued ASR genes *Yr1*, *Yr3a*, *Yr4a*, *Yr6*, *Yr7*, *Yr15*, *Yr17*, *Yr25*, *Yr27*, *Yr33*, and *Yr73* + *Yr74* were postulated as present singly or in various combinations in the remaining 73 cultivars (Online Resource Table S6). In many cases, resistance was detected that could not be attributed to specific ASR genes. The most commonly detected ASR gene was *Yr17*, postulated in 39 cultivars. This resistance gene occurs on the 2NS/2AS translocation along with the leaf rust resistance gene *Lr37* and stem rust resistance gene *Sr38*.

The seedling tests allowed not only postulation of catalogued ASR genes in the genotypes tested but also characterization of the seedling response of each to Pst687, which is avirulent for the *YrAvSAPR* resistance. One control genotype (Avocet R) and 62 cultivars were seedling resistant to Pst687 due to the presence of one or more catalogued (*Yr1*, *Yr15*, *Yr25*, *Yr27*, *Yr33*, *Yr73* + *Yr74*) or uncharacterized ASR genes (Online Resource Table S6). Nine control genotypes and 20 cultivars were seedling susceptible to Pst687 (Online Resource Table S6); adult plant tests confirmed or revealed the presence of *YrAvSAPR* in six of these control genotypes (Avocet S, Avocet S + Yr18, Avocet S + Yr29, Avocet S + Yr46, Lerma Rojo 64, Moomal 2000) and four of these cultivars (LRPB Hellfire, Sherriff CL Plus, Vixen, Wyalkatchem) (Online Resource Table S7). Based on low infection types to Pst687 that were higher than the “;” to “CN” typical of *YrAvSAPR*, three of the control genotypes and 12 cultivars that were seedling susceptible to Pst687 were postulated to lack *YrAvSAPR* (Online Resource Table S7). The status with respect to *YrAvSAPR* of the remaining four cultivars that were seedling susceptible to Pst687 was less clear due to the presence of APR to more than one pathotype (Online Resource Table S7). Two of these (Devil, Ninja) plus 11 other cultivars were considered to possibly carry *YrAvSAPR* based on the similarity of their responses to Pst687 to that recorded in Avocet S. Seven cultivars were also postulated to lack *YrAvSAPR* on this basis. The status of the remaining 42 cultivars with respect to *YrAvSAPR* could not be determined due to the presence of ASR genes that were effective against Pst687 (viz. *Yr25*, *Yr27*, *Yr33*, *Yr73* + *Yr74*; eight cultivars), uncharacterized ASR effective against Pst687 (six cultivars), or APR that was effective to at least one pathotype other than Pst687 (30 cultivars) (Online Resource Table S7).

## Discussion

*Pst* was first detected in Australia in 1979 (O’Brien et al. 1980). The pathotype responsible was identified as 104 E137 A-, and it was later shown to have likely originated from Europe (Ding et al. 2021). Three further incursions of *Pst* have been documented to date, pathotype 134 E16 A + in 2002 (Wellings 2007), 239 E237 A- 17 + 33 + in 2017, and 198 E16 A- J + T + 17 + in 2018 (Ding et al. 2021). Each of these incursions has impacted wheat production in Australia by rendering some resistant cultivars susceptible by virtue of virulence on one or more ASR genes. Evidence was also obtained for virulence on uncharacterized minor gene/APR following the 2002 incursion (Nazari 2006). The incursions of 2017 and 2018 resulted in changed stripe rust responses of many wheat cultivars being grown in eastern Australia. In some cases, changes in response could not be accounted for based on virulence on ASR genes, suggesting that the protective value of APR may have changed. Studies under controlled conditions to address this led to the discovery of a previously unknown gene conferring resistance to *Pst* at post-seedling growth stages.

The growth stage at which APR to rust in wheat is first expressed differs depending on the gene(s) responsible (Park and McIntosh 1994). Most studies characterizing APR to *Pst* in wheat have been based on field studies that have assessed stripe rust response at growth stages following flag leaf emergence. Field studies on the expression of APR to *Pst* in seven Australian wheat cultivars carrying genetically uncharacterized APR showed that expression of APR was more conspicuous during tillering to node formation (Park and Rees 1989). In the present study, inoculating plants 4 weeks after sowing at the 4 leaf growth stage (concurrent ZGS 14, 20) allowed identification of post-seedling resistance in some wheat genotypes when challenged with *Pst* pathotypes to which they were seedling susceptible. Under these experimental conditions, the minor APR genes *Yr18, Yr29* and *Yr46* singly conferred low levels or no resistance in most of the tests conducted. One post-seedling resistance discovered conferred a very high level of resistance that approached immunity, reminiscent of the hypersensitive type of resistance associated with many ASR genes. We mapped the gene conferring this resistance to the distal end of chromosome 2DS and designated it *Yr88*. Like many ASR genes, *Yr88* is race specific and is hence an example of a non-durable APR gene conferring resistance to *Pst*.

Wellings et al. (1988) reported that the cultivar Avocet was heterogeneous in its seedling response to two isolates of *Pst* collected in Australia in 1982 and 1983. Resistant (Avocet R) and susceptible (Avocet S) selections were established, and the latter used as a recurrent parent to develop a set of near isogenic stocks carrying single catalogued *Yr* genes. The resistance present in Avocet R was given the temporary designation *YrA* and was resolved as being conferred by two complementary genes (Wellings 1986). Based on rust isolate specificity, the resistance was also postulated to be present in both Australian (Banks, Condor, Avocet and Egret) and International (*e.g.* Anza, Nainari 60, Lerma Rojo 64, Inia 66, Sonalika) spring wheat cultivars, some of which were heterogeneous with respect to the *YrA* phenotype (Wellings et al. 1988). Genetic mapping later resolved the chromosomal locations of the two genes underlying the *YrA* specificity, and the genes were designated *Yr73* (chromosome 3DL) and *Yr74* (chromosome 5BL) (Dracatos et al. 2015). It was also demonstrated that while Avocet R carried both *Yr73* and *Yr74*, Avocet S carried *Yr73* only (Dracatos et al. 2015). The identification of *Yr88* in Avocet S, and its absence in Avocet R, provides a second example of difference in resistance to *Pst* between these two selections.

Nazari and El Amil (2013) reported the presence of ASR in Avocet S that was effective against three isolates of *Pst*, two collected from bread wheat and one from durum wheat in Syria in 2011. Detailed comparative greenhouse seedling tests provided strong evidence for the presence of the ASR in lines near isogenic to Avocet S carrying genes *Yr1*, *Yr5*, *Yr8*, *Yr10*, *Yr15*, *Yr17*, *Yr32*, and *YrSp*, and also in Avocet R, and its absence in Avocet S NILs carrying genes *Yr6*, *Yr7* and *Yr9*. The presence of *Yr88* in the Avocet S NILs carrying *Yr6*, *Yr7* and *Yr9*, and its absence in the Avocet S NIL carrying *Yr8* and in Avocet R indicate that this gene is different to the ASR reported by Nazari and El Amil (2013). Consistent with this are data of Hovmøller et al. (2022), which indicate that isolates within the PstS13 lineage to which Pst687 belongs (Ding et al. 2021) are virulent on seedlings of Avocet S and Avocet S NIL stocks carrying *Yr2*, *Yr6*, *Yr7*, *Yr8*, and *Yr9*.

Initial tests of Avocet S NIL stocks inoculated about 4 weeks after sowing established that *Yr88* was present in those carrying ASR genes *Yr6*, *Yr7*, *Yr9*, *Yr17* and the APR genes *Yr18* and *Yr29* but absent in the NIL stock carrying *Yr8*. This suggested a genetic relationship between *Yr88* and *Yr8*, the latter of which was transferred to hexaploid wheat from the grass species *Aegilops comosa* (Riley et al. 1968). The Avocet S + Yr8 NIL stock generated by Wellings et al. (2009) used the wheat line Compair as the donor of *Yr8*. Compair is the genotype used to represent gene *Yr8* in the European differential series of the differential set proposed by Johnson et al. (1972). It was developed by inter-crossing Chinese Spring with an accession of *Ae. comosa*, followed by a cross to *Ae. speltoides* to disrupt chromosome pairing control, then backcrossing to Chinese Spring (Riley et al. 1968). The absence of *Yr88* in the Avocet S + Yr8 NIL suggested that this gene may be located on a region of chromosome 2D that was lost during the integration of the *Ae. comosa* translocation carrying *Yr8*. Our mapping using a RIL population based on Avocet S confirmed this in locating *Yr88* to the distal region of chromosome 2DS.

Several studies have mapped APR to stripe rust in wheat to chromosome 2D, but in each case the resistances reported clearly differ from *Yr88*. Basnet et al. (2014) mapped a gene conferring APR to *Pst* to 2DL and designated it *Yr54*. Mustahsan et al. (2023) mapped a locus conferring field resistance in the cultivar Overland near to the telomere of 2DL. Li et al. (2024) reported a high temperature non-race-specific APR on the *Ae. comosa* translocated segment in the Avocet S + Yr8 NIL. Gene *Yr16*, first reported from the French wheat cultivar Cappelle Desprez with durable resistance to *Pst* (Lupton and Macer 1962), was mapped to chromosome 2D by Worland and Law (1986). Later studies mapped what is considered to be *Yr16* in the centromeric region of chromosome 2DS (Agenbag et al. 2012), and the positional and phenotypic evidence presented here supports the independence of *Yr88* and *Yr16*. The hypersensitive resistance conferred by *Yr88* differed from the minor level of resistance conferred by *Yr16* (Johnson 1984; Agenbag et al. 2012). The genotype Avocet S has been used as a susceptible parent to develop many populations to study APR to stripe rust in wheat. Some of these studies detected genomic regions associated with field resistance to *Pst* that was attributed to Avocet S, despite it being the stripe rust susceptible parent. These include QTL on chromosomes 4BL, 6AL, and 6BL (William et al. 2006), chromosome 2B (Rosewarne et al. 2008), chromosome 6AL (Lillemo et al. 2008), and chromosome 7 A (Rosewarne et al. 2012). In all cases, the resistance contributed by Avocet S was very minor and its expression in the populations was likely due to additivity with resistance/s coming from the second, resistant, parent. These QTL also differ from *Yr88* as none mapped to chromosome 2D.

Johnson (1992) conducted studies that compared the virulence of different isolates of *Pst* on adult plants and found evidence of four genes conferring APR based on differences in isolate specificity. These genes were designated *Yr11*, *Yr12*, *Yr13* and *Yr14*, but were not mapped to chromosomes. Virulence for each of these genes was detected after their deployment in wheat cultivars in the UK in the 1970 s, with major shifts in stripe rust responses that resulted in stripe rust epidemics in 1971/72 and 73 (*Yr11*; Joss Cambier), 1974/75/78/81 (*Yr13*; Maris Huntsman, Mardler or Hustler), and in 1978 (*Yr14*; Hobbitt) (Bayles 2001). It is possible that *Yr88* is one of the genes *Yr11*, *Yr12*, *Yr13* or *Yr14*. Heines VII, which carries *Yr11* (McIntosh et al. 1995), was resistant in adult plant tests with Pst687 (data not presented), however the additional presence of the ASR gene *Yr25* in Heines VII, for which Pst687 is avirulent, precluded any assessment of its pathogenicity on *Yr11*. The first incursion of *Pst* into Australia in 1979 was pathotype 104 E137, which was later shown to belong to MLG PstS0 (Ding et al. 2021). This pathotype was common in Europe in the 1970s, and for this reason Europe is suspected to be the likely origin of the isolate that appeared in Australia in 1979. In view of this, and the timeline, it is likely that the isolate of pathotype 104 E137 that was introduced into Australia carried virulence for one or more of the APR genes *Yr11*, *Yr12*, *Yr13*, and *Yr14*. If this is the case, and if *Yr88* is one of the genes *Yr11*–*Yr14*, it would explain why the APR gene in Avocet S was not detected with isolates of the original PstS0 incursion. Most of the cultivars that are designated stocks carrying one or more of the genes *Yr11*–*Yr14* also carry ASR genes that are effective against the *Yr88*-avirulent pathotype Pst687, meaning that the effectiveness of these APR genes cannot be assessed using these cultivars. Further research using either mutational derivatives of Pst687 with added virulence for the ASR genes present in cultivars carrying *Yr11*–*Yr14* that retain avirulence to *Yr88* or in genetic populations in which the effects of APR genes *Yr11*–*Yr14* can be separated from the ASR genes present, is required to assess the relationship of these genes with *Yr88*.

The detection of virulence in *Pst* for the APR gene *Yr88* in the present study represents the first example of virulence for a mapped gene conferring APR to *Pst* in wheat. Under the experimental conditions used in the present study, the slow rusting genes *Yr18* and *Yr29* conferred lower levels of resistance in comparison to *Yr88*, which conferred a strong hypersensitive-like resistant response to pathotypes with matching avirulence, similar to the resistant responses conferred by the non-durable catalogued genes *Lr12* and *Lr22b* that confer APR to leaf rust in wheat. Virulence for both *Lr12* and *Lr22b* has been detected in Australia, where it appears to have been common (Park and McIntosh 1994). It is likely that *Yr88*, *Lr12* and *Lr22b* differ in action to slow rusting genes like *Yr18*, *Yr29* and *Yr46*. Two of the latter genes have been cloned, shedding light on the way in which they confer resistance. *Yr18*/*Lr34*/*Sr57* encodes an ATP-binding cassette transporter (Krattinger et al. 2009) and *Lr67*/*Yr46*/*Sr55* encodes a protein that has lost hexose transport function (Moore et al. 2015). Both confer resistance against all three rust pathogens and are associated with a linked morphological trait known as leaf tip necrosis. Experience to date suggests that these pleiotropic APR genes have some intrinsic durability, and that combinations of multiple effective resistance genes contribute to durability by lowering the chance of virulence matching gene combinations developing. In many instances, pleiotropic APR genes have been used to great effect in conjunction with ASR genes, providing backbone resistance that may also contribute to enhancing the durability of co-deployed ASR genes (Park 2015).

Exotic wheat rust isolates that have entered Australia have sometimes carried avirulence for resistance genes that were previously ineffective, meaning that wheat cultivars carrying such genes were resistant to the new pathotype despite being formerly susceptible (e.g., *Lr73*, Park et al. 2014). The APR gene discovered in the studies reported here was only effective against two of the pathotypes used, viz. Pst687 and Pst698. Pst687 is an isolate of pathotype 198 E16 A- J + T + 17 +, first detected in Australia in 2018. Microsatellite genotyping established that this pathotype belongs to the multi-locus genotype (MLG) group PstS13, having originated from either Europe or South America (Ding et al. 2021). Pst698 is an isolate of pathotype 238 E191 A- J + T + 17 +, first detected in Australia in 2021, which is considered a single-step mutational derivative of Pst687 with added virulence for the ASR gene *Yr25* (Park RF unpublished).

The first locus conferring resistance to *Pst* in wheat was mapped and designated as *Yr1* in 1962 (Lupton and Macer 1962). Of the 25 loci conferring APR to *Pst* mapped and designated up to 2025, 17 were catalogued between 2011 (*Yr46*; Herrera-Foessel et al. 2011) and 2023 (*Yr86*; Zhu et al. 2023) reflecting increasing interest in characterizing and using APR to control *Pst* in wheat. Previous reports of race-specific APR to *Pst* in wheat from Europe (Stubbs 1985), the UK (Johnson 1992), and the USA (Milus et al. 2015) suggest that this type of resistance is not uncommon in the global hexaploid wheat germplasm pool. Our results provided evidence of *Yr88* not only in four Australian wheats but also in the Mexican wheat Lerma Rojo 64 and the Pakistani wheat cultivar Moomal 2000. Lerma Rojo 64 (Yaqui 50//Norin 10/Brevor/3/Lerma 52/4/2*Lerma Rojo) was bred at the International Wheat and Maize Improvement Centre (CIMMYT) in Mexico and was foundational in the green revolution in India, Pakistan and other countries (Lumpkin 2015). Interestingly, Lerma Rojo 64 is considered the likely ancestral source of the *Yr73* + *Yr74* complementary resistance to stripe rust in several Australian wheat cultivars including Avocet (Wellings et al. 1988). The characterization of *Yr88* will assist in understanding pathogenic variability in *Pst* on APR, and importantly in avoiding the selection of non-durable sources of APR to *Pst* in wheat improvement.

## Supplementary Information

Below is the link to the electronic supplementary material.

Supplementary file1 (PPTX 74 KB)

Supplementary file2 (XLSX 84 KB)

## Data Availability

The datasets generated during and/or analyzed during the current study are available from the corresponding author on reasonable request.
